# Optimizing flexible and navigable ureteral access sheaths (FANS) manoeuvrability: the impact of sheath size, working channel occupancy and deflection technique – an EAU endourology in vitro analysis

**DOI:** 10.1186/s12894-026-02281-4

**Published:** 2026-07-27

**Authors:** Steffi Kar Kei Yuen, Vineet Gauhar, Alex Qinyang Liu, Ivan Ching Ho Ko, Yuen Hei Chung, Jia-Lun Kwok, Chi-Ho Leung, David Ka-Wai Leung, Francis Chun Him Wong, Joseph Kai Man Li, Hoi Chak Chan, Yi Quan Tan, Arman Tsaturyan, Bhaskar Somani, Chi-Fai Ng

**Affiliations:** 1 S.H. Ho Urology Centre, Department of Surgery, The Chinese University of Hong Kong, Hong Kong, China; 2 European Association of Urology Section of Endourology (ESEUT), Arnhem, The Netherlands; 3 Division of Urology, Ng Teng Fong General Hospital, Singapore, Singapore; 4 Asian Institute of Nephrology and Urology, AINU, Hyderabad, India; 5 Department of Urology, Tan Tock Seng Hospital, Singapore, Singapore; 6 Lee Kong Chian School of Medicine, Nanyang Technological University, Singapore, Singapore; 7 Department of Surgery, Tuen Mun Hospital, Hong Kong, China; 8 Department of Urology, National University Hospital, Singapore, Singapore; 9 Department of Urology, Erebouni Medical Center, Yerevan, Armenia; 10 Department of Urology, Yerevan State Medical University, Yerevan, Armenia; 11 Department of Urology, University Hospital Southampton, NHS Trust, Southampton, UK

**Keywords:** Flexible ureteroscopy, Flexible and navigable suction ureteral access sheaths, Suction, Deflection, Urolithiasis

## Abstract

**Introduction:**

Conventional ureteral access sheaths are placed below the ureteropelvic junction (UPJ), whereas the novel flexible and navigable suction ureteral access sheaths (FANS) are navigated across the UPJ with its distal tip deflected into target calyx under endoscopic guidance to facilitate stone fragments suctioning. We evaluate how FANS deflection angles are affected by sheath sizes, occupancy of working channel (WC) (empty, occupied by Olympus Soltive™ Thulium Laser Fiber 150 μm and 200 μm core diameter) and deflection techniques (ureteroscope deflection versus sheath advancement) across four brands of FANS.

**Materials & methods:**

In-vitro deflection angles of brand new FANS (sheath sizes 10/12Fr, 11/13Fr and 12/14Fr) ClearPetra (Well Lead Medical Co., Ltd, China) 46 cm, Elephant II (YiGao Medical Technology Co., Ltd, China) 50 cm, Innovex (Innovex Medical Co., Ltd, Shanghai, China) 50 cm, and Reborn (Hunan Reborn Medical Science and Technology Development Co., Ltd, China) 50 cm with brand new single-use flexible ureteroscope Pusen Uscope 7.5Fr PU3033A (Zhuhai Pusen Medical Technology Co., Ltd. Guangzhou, China) were measured in triplicate with varied WC occupancy and forementioned techniques.

Maximum deflection angle was expressed as a mean of 3 measurements. One-way ANOVA test was performed for comparisons of the effect of sheath sizes, sheath brands, working channel occupancy, and different deflection techniques. Four-way ANOVA test was performed to evaluate the influence of the aforementioned four factors on the maximal deflection angle, with analysis on the main effects and interaction. For all tests, a two-sided p-value less than 0.05 was considered statistically significant. Statistical tests were performed with SPSS version 26.0 (IBM Corp., Armonk, NY, USA).

**Results:**

10/12Fr and 11/13Fr sheath sizes have significantly better deflection angles than 12/14Fr across all brands with a 7.5Fr flexible ureteroscope. Sheath advancement technique was significantly better with ureteroscope deflection technique. There is no significant difference in deflection angles with occupancy of 150–200 μm fiber.

**Conclusion:**

Gaining better understanding of the performance of FANS can ultimately guide clinicians in their selection and optimal utilization of the instrument to enhance procedural efficacy and treatment outcomes.

## Introduction

Conventional ureteral access sheaths are placed below the ureteropelvic junction (UPJ), whereas flexible and navigable suction ureteral access sheaths (FANS) are navigated across the UPJ with its distal tip deflected into target calyx under endoscopic guidance to facilitate stone fragments suctioning [1–3].

Lower pole stones have been a challenge for flexible ureteroscopy (fURS) in terms of access and complete removal of stone fragments. Shrestha et al. have shown that the use of smaller ureteroscopes, thulium fiber laser (TFL) and suction technology can help overcome the challenges with the infundibulopelvic angles, improving fURS access and stone free rates (SFRs) [4]. Suction via FANS has shown feasible even in a deflected position, increasing SFRs, reducing usage of stone basket, improving patient quality of life with minimal infectious complications [5–7].

The above advantages are dependent on optimal deflection of the flexible pliable and atraumatic FANS tip. With several manufacturers in the market, it is important to review the actual physical deflection properties of FANS. This will facilitate the choice of sheath brand and sizes, laser fiber and deflection technique in each patient. Previously, studies have shown that advertised physical and mechanical properties are often not the same in actual experimental studies both for ureteral access sheaths and fURS [8–11]. A recent study has demonstrated optimal FANS deflection techniques, namely the sheath advancement technique, and sheath size-ureteroscope combinations, but only across one brand of FANS [12]. The paucity of knowledge with different FANS deflection abilities hinders the ability to optimize the choice of FANS in clinical practice, a critical consideration as such suction devices become more widely adopted [13]. Gaining better understanding of the performance of FANS will ultimately guide clinicians in their selection of the instrument to enhance procedural efficacy and patient outcomes.

This study aims to evaluate how FANS deflection angles across four sheath brands are affected by (1) sheath sizes, (2) occupancy of the working channel (WC) and (3) deflection techniques.

## Materials and methods

We evaluated in-vitro deflection angles of three different FANS sizes across four different brands of FANS with the single-use flexible ureteroscope Pusen Uscope 7.5Fr PU3033A (Zhuhai Pusen Medical Technology Co., Ltd. China) at the Chinese University of Hong Kong.

**Fig. 1:**
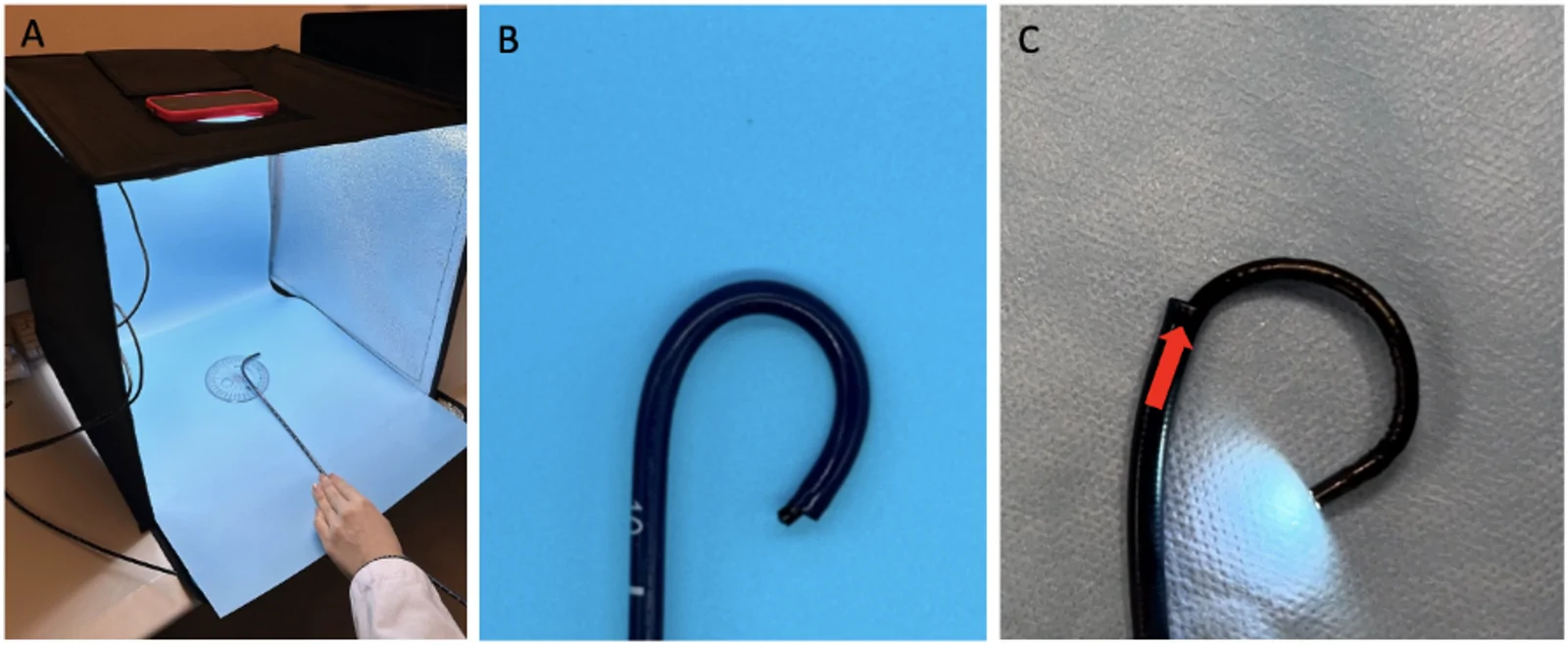
**A** Experimental setup. **B** Ureteroscope deflection technique. **C** Sheath advancement technique (red arrow indicates direction of sheath advancement over a deflected scope tip)

Brand new FANS available at the Chinese University of Hong Kong were used in the study, including manufacturer labelled sheath sizes 10/12Fr, 11/13Fr and 12/14Fr of ClearPetra (Well Lead Medical Co., Ltd, China) 46 cm, Elephant II (YiGao Medical Technology Co., Ltd, China) 50 cm, Innovex (Innovex Medical Co., Ltd, China) 50 cm, and Reborn (Hunan Reborn Medical Science and Technology Development Co., Ltd, China) 50 cm. All ureteroscopes used were reverse deflection models. The ureteroscopes were tested under the same experimental setup with a fixed lightbox and iPhone15 camera (Fig.1 A). Prior to each measurement, the ureteroscope was checked by three independent authors (DKWL, CHW, KML) to ensure intact quality of deflection mechanism and that the maximal deflection angle achieved were per manufacturer specifications. The ureteroscopes were replaced with new ones at any point if the deflection mechanism was impaired.

After the ureteroscope was introduced into FANS, the straight axis of FANS and ureteroscope were aligned to the axis of the fixed goniometer in the camera lightbox (Fig. 1A). A camera held at a fixed height to capture the maximum deflection angles. Measurements were taken only in maximum downward deflection, as the downward and upward deflection angles stated by the manufacturers were the same for the ureteroscope.

**Fig. 2:**
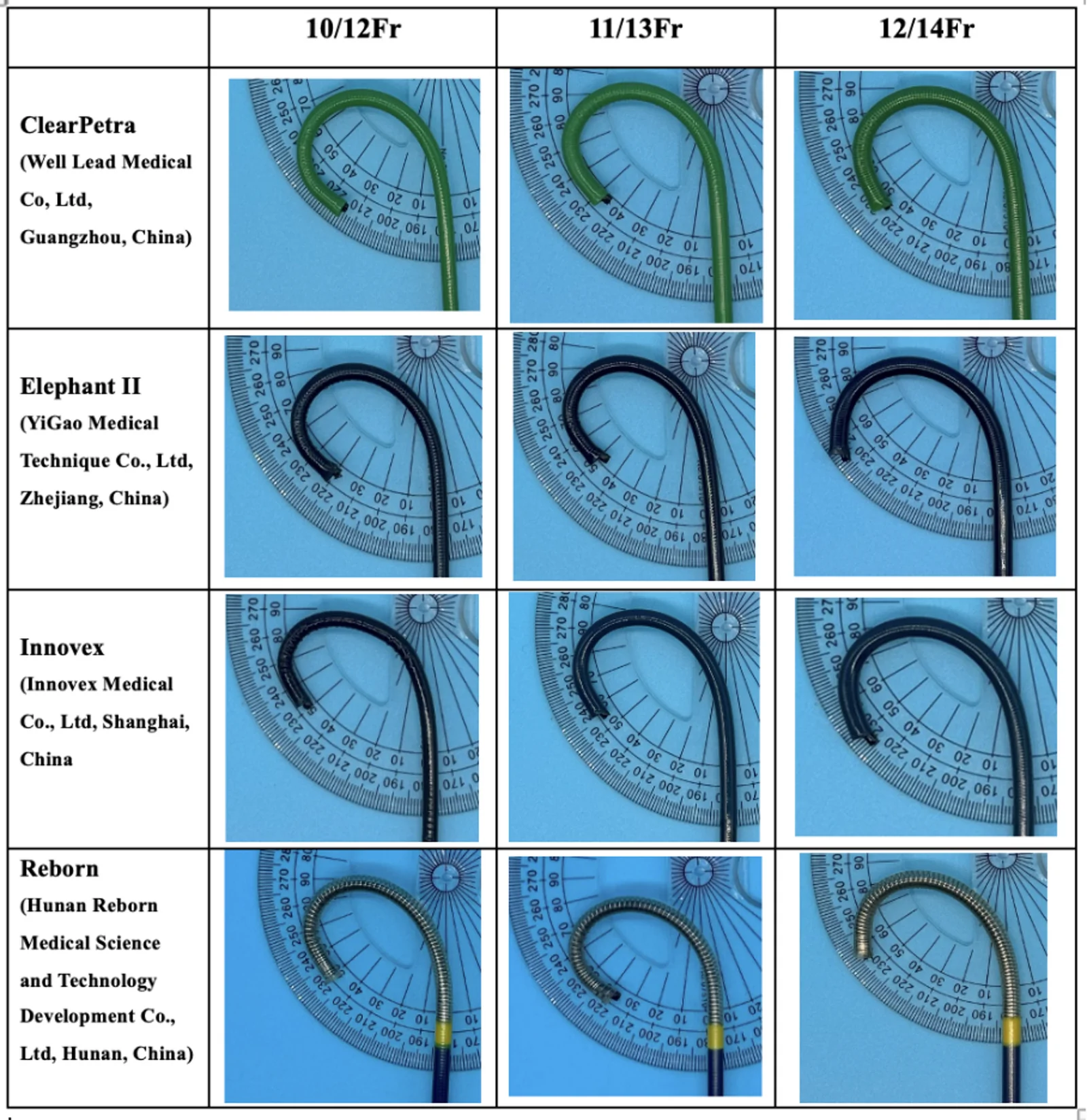
Maximum deflection angles across different sheath sizes (10/12Fr, 11/13Fr and 12/14Fr), using ureteroscope deflection technique, with no laser fibre, stratified by sheath brands

Measurements were taken with the ureteroscope WC being either (1) empty; or occupied with a (2) 150 μm Soltive TFL; or (3) 200 μm Soltive TFL. The following deflection techniques were tested, as previously defined [12] (1) ureteroscope deflection technique – The ureteroscope is placed within the FANS sheath, which is then maximally deflected to actively bend the FANS sheath. How it works: Begin with the flexible tip of the ureteroscope straight, positioned just at the edge of the FANS and then bend (deflect) the tip of the scope as far as it will go to negotiate the sheath and ureteroscope together. (Fig. 1B); (2) sheath advancement technique – the scope is deflected first, then slide the FANS sheath over the deflected scope tip. How it works: First, extend the flexible part of the ureteroscope past the sheath and deflect (bend) the tip downward fully. Then, while maintaining the deflected scope tip, slide the FANS forward over the deflected scope tip, using the ureteroscope as a endoscopic guide for the sheath (Fig. 1C).

Deflection angles were measured in triplicate for each of the above deflection techniques and each working channel occupancy situation. The maximum deflection angle of the distal tip of the access sheath was measured using the software Adobe Photoshop 2024 (25.11.0 Release) solely by one of the authors(ICHK).

### Statistical analysis

For each measurement, the maximum deflection angle was expressed as a mean of three measurement readings. One-way ANOVA test was performed for comparisons of the effect of sheath sizes, sheath brands, working channel occupancy, and different deflection techniques. Four-way ANOVA test was performed to evaluate the influence of the aforementioned four factors on the maximal deflection angle, with analysis on the main effects and interaction. For all tests, a two-sided p-value less than 0.05 was considered statistically significant. All statistical tests were performed with SPSS version 26.0 (IBM Corp., Armonk, NY, USA).

## Results

The mean maximal deflection angles under all included test conditions are summarized in Table 1. The table charts the deflection across different manoeuvrability characteristics — specifically ureteroscope deflection and sheath advancement — of four commercially available flexible ureteric access sheaths (ClearPetra, Elephant II, Innovex, and Reborn) across three sheath sizes (10/12Fr, 11/13Fr, 12/14Fr) and three working channel conditions (no laser, 150 μm fiber, 200 μm fiber).

**Table 1:** Mean maximal deflection angles of FANS with single-use flexible ureteroscope across four experimented brands with different sizes, working channel occupancy and deflection techniques

Inner size	10/12Fr		11/13Fr		12/14Fr			
Technique	Ureteroscope deflection	Sheath advancement	Ureteroscope deflection	Sheath advancement	Ureteroscope deflection	Sheath advancement	ANOVA of Sheath advancement (intra-sheath)	ANOVA of Ureteroscope deflection (intra-sheath)
	ClearPetra (Well Lead Medical Co, Ltd, Guangzhou, China)							
No laser	154.1	222.8	144.0	221.5	134.0	209.4	*p* = 0.36	*p* = 0.60
150 μm	152.2	220.8	151.2	220.9	127.3	201.7	*p* = 0.27	*p* = 0.28
200 μm	170.3	224.4	152.7	216.1	132.2	194.9	*p* = 0.12	*p* = 0.18
ANOVA of all WC intrasheath	*p* = 0.55	*p* = 0.94	*p* = 0.90	*p* = 0.91	*p* = 0.91	*p* = 0.41		

	Elephant II (YiGao Medical Technique Co., Ltd, Zhejiang, China)							
No laser	153.0	229.3	114.1	216.3	97.6	171.5	*p* = 0.05	*p* = 0.10
150 μm	155.2	226.5	139.7	200.4	104.3	182.2	*p* = 0.17	*p* = 0.02
200 μm	159.2	219.4	139.1	199.7	98.6	171.5	*p* = 0.11	*p* = 0.01
ANOVA of all WC intrasheath	*p* = 0.87	*p* = 0.66	*p* = 0.44	*p* = 0.70	*p* = 0.85	*p* = 0.87		

	Innovex (Innovex Medical Co., Ltd, Shanghai, China)							
No laser	122.7	204.6	139.1	219.9	143.5	218.3	*p* = 0.36	*p* = 0.53
150 μm	115.9	193.2	147.1	210.9	159.8	218.3	*p* = 0.25	*p* = 0.07
200 μm	123.1	204.6	159.0	216	154.9	205.8	*p* = 0.78	*p* = 0.15
ANOVA of all WC intrasheath	*p* = 0.85	*p* = 0.51	*p* = 0.52	*p* = 0.78	*p* = 0.72	*p* = 0.80		

	Reborn (Hunan Reborn Medical Science and Technology Development Co., Ltd, Hunan, China)							
No laser	117.0	202.9	136.2	216.6	86.8	162.5	*p* = 0.01	*p* = 0.08
150 μm	121.8	207.3	124.2	212.5	109.3	185.5	*p* = 0.17	*p* = 0.59
200 μm	119.3	200.1	151.5	217.9	119.5	184.4	*p* = 0.07	*p* = 0.03
ANOVA of all WC intrasheath	*p* = 0.90	*p* = 0.92	*p* = 0.38	*p* = 0.83	*p* = 0.13	*p* = 0.10		

### Inter-sheath comparison at baseline

With an emptying WC, ClearPetra demonstrated the highest mean maximum deflection angles across all sheath sizes, followed closely by Innovex and Elephant II, with Reborn exhibiting the lowest angles. ANOVA testing showed statistically significant inter-brand differences for both ureteroscope deflection and sheath advancement techniques in the absence of laser fiber (*p* < 0.001).

### Effect of sheath size

There was a wide range of maximal deflection angles achieved across 10/12Fr, 11/13Fr and 12/14Fr, ranging from 117°-154°, 114°-144°, 87°-143°, respectively. Figure 2 illustrates the angles across different sheath sizes. 10/12Fr and 11/13Fr had better maximal deflection angles than 12/14Fr across Clearpetra, Elephant II and Reborn, demonstrating that smaller sheath sizes were generally associated with higher maximum deflection angles across all brands, with performance declining as sheath size increased to 12/14Fr. The exceptions being Innovex sheaths having better deflection angles in 12/14Fr compared to its 10/12Fr counterpart, and Reborn sheaths of size 11/13Fr performing better than 10/12Fr. Statistical significance was reached for Elephant II (sheath advancement technique without laser, and ureteroscope deflection technique with laser fiber) and Reborn (no laser and 200 μm fiber). There is also definite variable trend in terms of deflection magnitude between brands.

### Effect of working channel occupancy

**Fig. 3:**
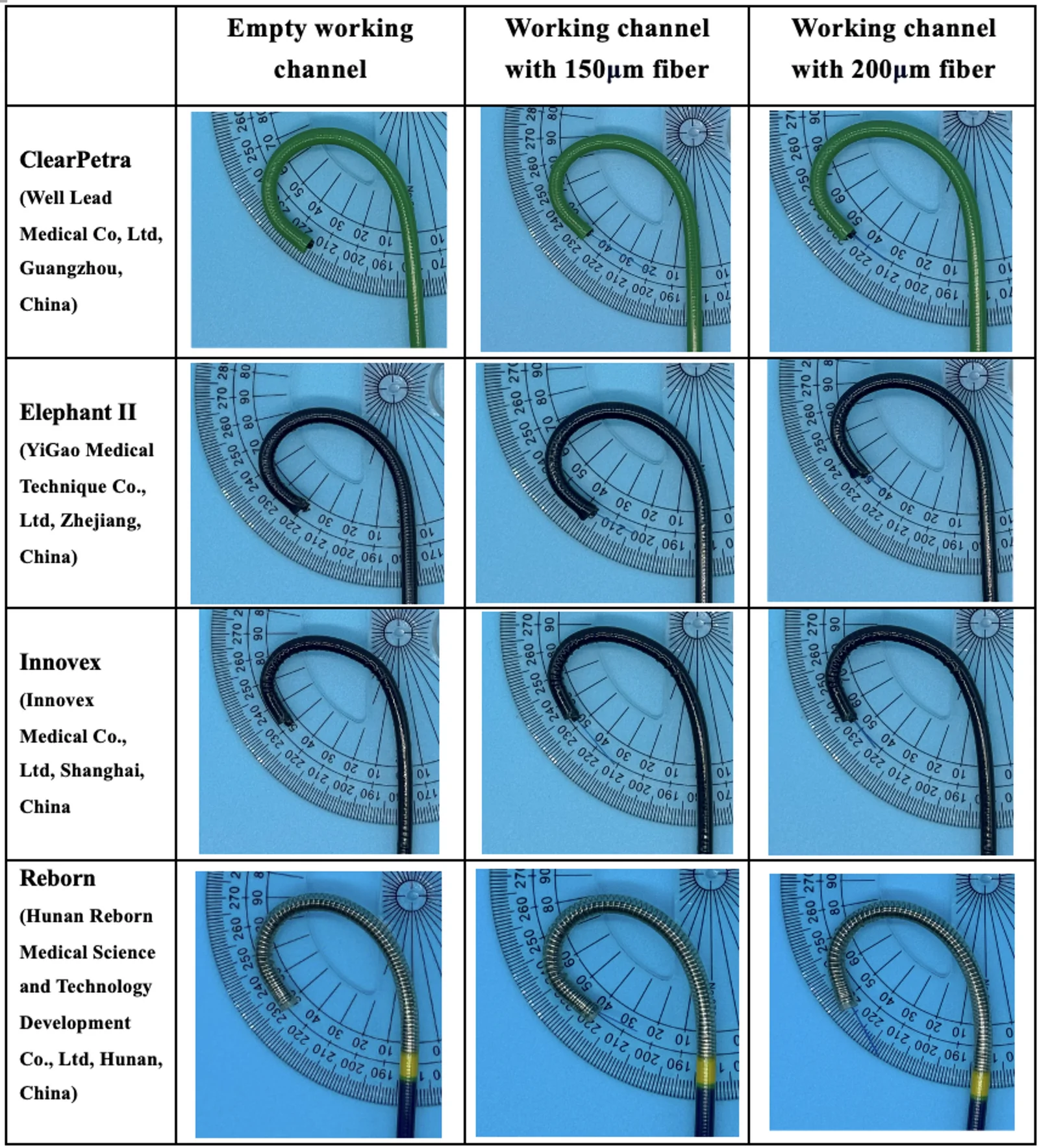
Maximum deflection angles across different working channel occupancy (no laser, 150 μm laser, and 200 μm laser), using ureteroscope deflection technique, with 10/12Fr sheath, stratified by sheath brands

Figure 3 shows the deflection angles across different working channel occupancy conditions (no laser, 150 μm fiber, and 200 μm fiber). All sheaths maintained relatively stable performance across all working channel conditions, with no statistically significant intra-brand differences across different occupancy for either deflection or advancement (ANOVA *p* > 0.05). There is better maximal angle deflection with laser occupying the working channel, though not statistically significant.

### Effect of deflection technique

**Fig. 4:**
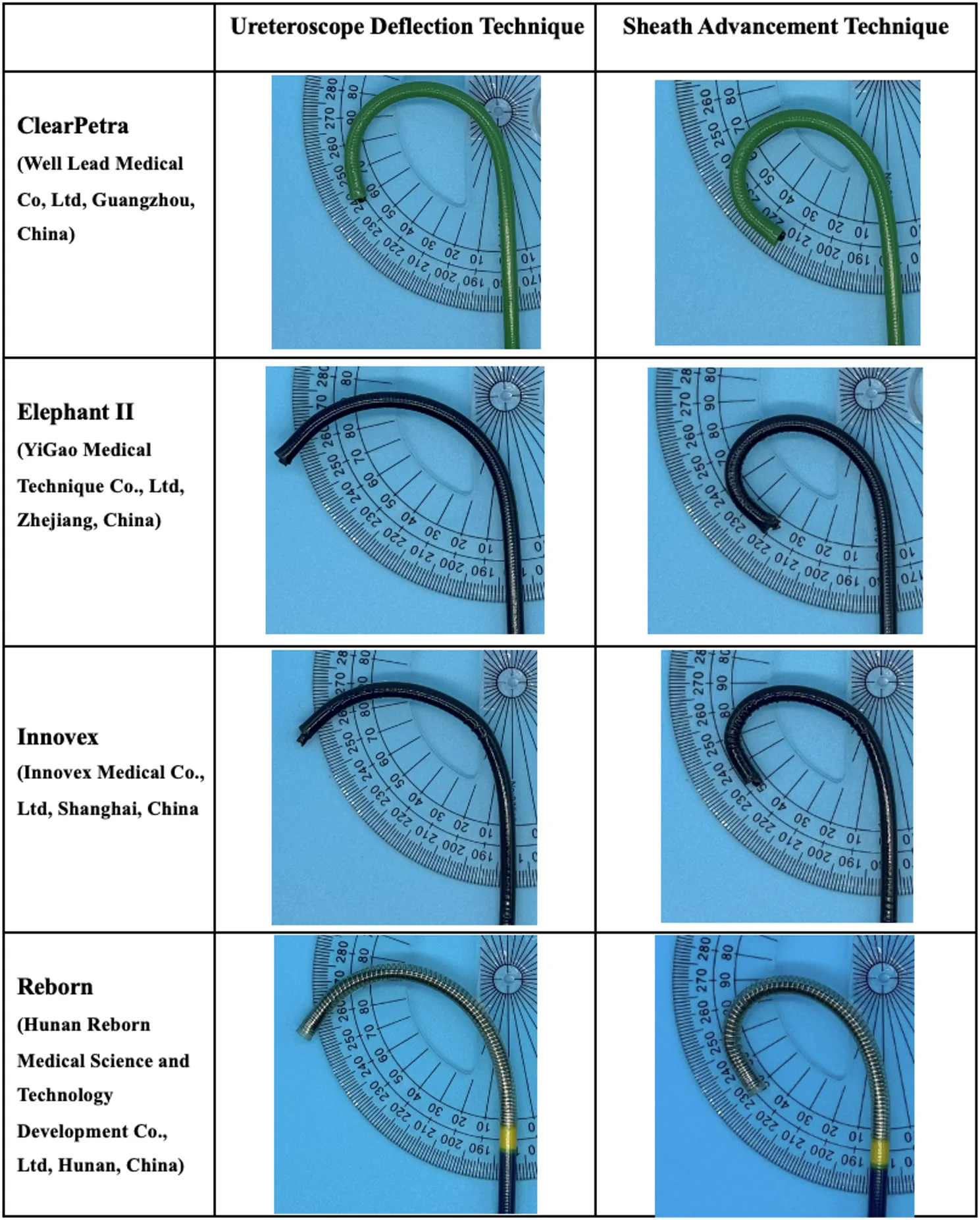
Maximum deflection angles across different techniques (ureteroscope deflection and sheath advancement), with 10/12Fr sheath and no laser, stratified by sheath brands

Across all brands and conditions, the sheath advancement technique consistently produced greater maximal deflection angles than ureteroscope deflection technique. Figure 4 shows the deflection angles achievable by the two different techniques across different sheath brands. With the sheath advancement technique, the largest mean maximum deflection angles were achieved with ClearPetra (194.9–224.4º), followed by Innovex (193.2–219.9º), Elephant II (171.5–229.3), and lastly Reborn (162.5–216.6º). In comparison, with the ureteroscope deflection technique, ClearPetra again exhibited the a lower maximal angle (127.3–170.3°), followed by Innovex (115.9–159.8º), Elephant II (97.6–159.2º), and Reborn (86.8–151.5º). Statistical comparison confirmed a significant difference between the two techniques across all brands and sheath sizes (*p* < 0.001).

### Four-way ANOVA test

A four-way ANOVA, as shown in Table 2, was conducted to assess the effects of the four variables, namely brand, working channel occupancy, sheath size, and deflection technique on maximal deflection angle. The analysis revealed that brand, sheath size, and deflection technique each had statistically significant effects. Specifically, brand significantly influenced deflection angle (F = 13.41, *p* < 0.001), indicating notable performance differences among the ureteric access sheath manufacturers. Sheath size also had a significant effect (F = 25.54, *p* < 0.001), with smaller sized sheaths generally permitting greater deflection. The deflection technique used — ureteroscope deflection versus sheath advancement — had the most pronounced impact on deflection angle (F = 783.21, *p* < 0.001). In contrast, working channel occupancy, reflecting the presence or absence of a laser fiber (none, 150 μm, or 200 μm), did not significantly affect the maximal deflection angle (F = 0.81, *p* = 0.447).

**Table 2:** 4-way ANOVA table with brand, working channel occupancy, sheath size, and technique as independent variables

Factor	df	F	*P*
Brand	3	13.41	< 0.001
Working channel occupancy	2	0.81	0.447
Sheath size	2	25.54	< 0.001
Technique	1	783.21	< 0.001
Brand * working channel occupancy	6	0.48	0.822
Brand * sheath size	6	13.47	< 0.001
Brand * technique	3	0.74	0.528
Working channel occupancy * sheath size	4	0.60	0.666
Working channel occupancy * technique	2	2.75	0.067
Sheath size * technique	2	0.26	0.775
Brand * working channel occupancy * sheath size	12	0.68	0.765
Brand * working channel occupancy * technique	6	0.21	0.974
Brand * sheath size * technique	6	0.55	0.770
Working channel occupancy * sheath size * technique	4	0.29	0.882
Brand * working channel occupancy * sheath size * technique	12	0.24	0.996

Among the interaction effects, only the interaction between brand and sheath size reached statistical significance (F = 13.47, *p* < 0.001), suggesting that the influence of sheath size on deflection performance differed across brands. All other two-way, three-way, and four-way interactions—including those involving working channel occupancy and technique — were not statistically significant (all *p* > 0.05), indicating that these variables primarily operated independently without meaningful interaction effects.

## Discussion

The study aimed to characterize the deflection angles offered by the current generation of FANS sheaths commercially available. Relaying information on factors affecting FANS deflection angles could guide clinical practice. A deflection angle of 104° to 175° is often required of a flexible ureteroscope to access the lower-pole infundibulum [14], such requirements also translate into those of FANS.

### Sheath size matters

This is central to FANS utilization and, although RESD [15] plays a role, the deflection characteristics of the sheath can impact operative time and scope lifespan.

### Smaller is generally better

In line with earlier findings [16], using a 7.5Fr flexible ureteroscope with smaller (10/12Fr and 11/13Fr) FANS, generally demonstrated superior deflection angles when compared to larger 12/14Fr sheaths. Advances in materials have likely made newer, smaller sheaths easier to manoeuvre since they require less deflection force, improving navigation in the pelvicalyceal system(PCS). Early models had deflection angles around 110° to 130° on their own, which dropped significantly to about 90° to 130° when loaded with instruments [17].

### Impact of technique: sheath advancement versus ureterscope deflection

The sheath advancement technique effectively addresses challenging angles that may not be accessible through ureteroscope deflection alone. Across all brands evaluated, when ureteroscope deflection was insufficient, employing sheath advancement resulted in significantly improved maximum deflection angles. Optimising deflection within the PCS is crucial for accessing its various regions and facilitating stone management. The findings presented here suggest important clinical applications: when active deflection does not allow access to the stone, advancing the sheath over the scope provides a practical alternative, permitting the laser fibre to reach the stone surface and enabling controlled in-situ dusting and aspiration of fragments. This approach can eliminate the need for stone relocation. Furthermore, these insights may help prevent scope damage, as practitioners informed by our results can avoid excessive manipulation of control levers—particularly important for multi-use reusable scopes that may already exhibit wear.

### Brand variations

Understanding brand variations enables clinicians to make informed decisions based on the degree of hydronephrosis, size of the PCS, shape of the UPJ, and potentially different considerations for adult versus pediatric cases. This awareness also highlights opportunities for developing paediatric-specific devices for fURS, a topic frequently discussed in the literature [18] Notably, ClearPetra and Elephant II have shown superior deflection in smaller sizes (10/12Fr and 11/13Fr), whereas Innovex demonstrated enhanced deflection in the larger size (12/14Fr).

### Working channel occupancy

The WC has been discussed in relation to fluid and laser fibre impact [19]. In fact, too small a scope, weaker shaft paired with a bigger diameter sheath may further limit the reach of the sheath to the desired calyx. Proper knowledge of sheath dynamics can help subvert this. Interestingly, the presence of Thulium Fiber Laser (TFL) fibres (150 μm vs. 200 μm) did not mechanically interfere with sheath deflection in our in vitro study. Perhaps in clinical studies where TFL was adopted with better Zero Residual Fragment (ZRF) status could also be in part due to sheath properties albeit this was only indirectly reported using a Likert scale evaluation [20]. The implied findings of our study can allow surgeons to choose fibre diameter based on lithotripsy needs without worrying about compromising sheath manoeuvrability when paired with a good sheath deflection. This could also be a potential area of research as many types of laser machines have evolved.

### Clinical implications and limitations

Surgeons must anticipate the range of movement when using different sheath sizes. To optimise surgical strategy whereby in FANS the fundamental principles involve reaching the stone with the flexible ureteroscope, dust in situ to reduce stone burden and advance the sheath appropriately to aspirate. This emphasizes the need for sheaths with high-quality hydrophilic coatings and durable flexible tips to prevent mucosal injury. As we understand, fluid dynamics when using FANS proper equipment selection and deflection angles affects irrigation/suction ratios that remain a critical factor to consider alongside mechanical deflection [3, 21]. For this study we have only investigated on one scope size, in an open environment without the PCS elasticity and active fluid flow that can cause further impedance to sheath deflections. We chose 7.5Fr as that is the most common ureteroscope available in our locality and set up. Perhaps smaller and larger size scopes may have a difference in deflection strength, and hence deflection angles of FANS and will make for an interesting comparison [12].

Future clinical studies are required to validate our in vitro findings, as well as investigate the relationship with different flexible ureteroscope sizes and brands, optimal FANS sheath length, lasing techniques, and optimal fluid dynamics - all that may affect the outcomes of FANS ureteroscopy.

## Conclusion

This study demonstrates that FANS deflection is significantly influenced by sheath size and deflection technique, with smaller sheath sizes generally offering superior manoeuvrability across most brands. Crucially, sheath advancement technique yields significantly greater maximum deflection angles compared to standard active ureteroscope deflection. Furthermore, the presence of TFL fibres within the working channel does not mechanically impede deflection, simplifying fibre selection for surgeons. While these in vitro findings provide a critical framework for preoperative planning and equipment selection, future clinical studies integrating fluid dynamics and varying scope sizes are necessary to validate these results in vivo.

## Data Availability

The data that support the findings of this study are available from the corresponding author upon reasonable request.
